# Recognition of phrenic paralysis as atypical presentation during CT chest examination of COVID-19 infection and its correlation with CT severity scoring: a local experience during pandemic era

**DOI:** 10.1186/s43055-021-00527-9

**Published:** 2021-06-23

**Authors:** Emad H. Abdeldayem, Ahmed S. Abdelrahman, Mohamed G. Mansour

**Affiliations:** Radiology Department, Faculty of Medicine, Ain Shams University, Cairo, Egypt

**Keywords:** CT severity score, COVID-19 pneumonia, Phrenic nerve paralysis, RT-PCR

## Abstract

**Background:**

Coronavirus disease 2019 (COVID-19) was declared a global pandemic by the World Health Organization on March 11, 2020. COVID-19 infection is considered a multi-system disease with neurological, digestive, and cardiovascular symptoms and complications. It can trigger acute and diffuse endothelial dysfunction, resulting in a cytokine storm, most likely induced by the interleukin-6 (IL-6) amplifier. The peripheral and central neurological complications may explain some clinical manifestations such as vagus nerve palsy. The known main CT chest findings of COVID-19 pneumonia include ground glass patches, pulmonary consolidations, inter-lobar septal thickening, crazy paving appearance, and others. We presented our experience in the incidental discovery of phrenic nerve paralysis as atypical chest finding in patients with a known history of COVID-19-associated pneumonia, proved by RT-PCR and coming for evaluation of the lung changes. Patients with evidence of diaphragmatic paralysis underwent close follow-up with a re-evaluation of the phrenic nerve palsy at their routine follow-up for COVID-19 pneumonia. The association of the phrenic nerve palsy was correlated with the CT chest severity score.

**Results:**

Among 1527 scanned patients with known COVID-19 pneumonia, we had recognized 23 patients (1.5%) with unilateral diaphragmatic paralysis, accidentally discovered during CT chest examination. Twenty-one patients had shown complete recovery of the associated diaphragmatic paralysis during their follow-up CT chest with regression or the near-total resolution of the pulmonary changes of COVID-19- pneumonia. No significant correlation between the incidence of unilateral diaphragmatic paralysis and CT severity score with p value = 0.28.

**Conclusion:**

Phrenic paralysis is considered a serious but rare neurological complication of COVID-19 pneumonia. No significant correlation between the CT severity score and the incidental discovery of unilateral diaphragmatic paralysis. The majority of the cases show spontaneous recovery together with the improvement of the pulmonary changes of COVID-19 pneumonia. The association of phrenic paralysis with anosmia and dysgeusia could suggest a direct viral attack on the nerve cells.

## Background

COVID-19 is an infectious disease of the respiratory system caused by (SARS-CoV-2), which is a strain of coronavirus. COVID-19 usually presents with clinical manifestations related to pneumonia [1]. The clinical presentation of COVID-19 infection varies from asymptomatic cases up to acute respiratory distress syndrome (ARDS) [2]. The spectrum of infection extends to include gastrointestinal and neurological complaints. It has been considered a pandemic crisis by the WHO in March 2020 [3, 4]. The high rate of false-negative results of RT-PCR, particularly early in the course of the disease process, and the inconsistent availability of testing, necessitate a systematic approach to diagnosis, including the use of radiologic imaging [5–7]. Imaging plays an important role in the detection and follow-up of cases with COVID-19 infection. CT chest examination is considered the preferred imaging tool for cases with clinical suspicion of COVID-19 as well as evaluation of the degree of pulmonary affection [8, 9]. Phrenic paralysis should be considered in cases of COVID-19 pneumonia when orthopnoea and paradoxical abdominal respiration are present [10]. The virus may attack the olfactory nerve cells resulting in anosmia and dysgeusia. Moreover, SARS-CoV-19 may invade peripheral nerve terminals and such findings could explain the phrenic paralysis in COVID-19 patients, resulting in pulmonary decompensation [11–13].

The present study aimed to focus upon incidental detection of the diaphragmatic paralysis in known COVID-19 patients and correlate the incidence with the CT chest severity score. Also, our study aims to detect the prognosis and recovery of the phrenic paralysis together with the time interval changes of the pulmonary changes of COVID-19 pneumonia.

## Methods

### Patients

This cross-sectional study was done in the period from the first of May till 30 November 2020 and included 1527 patients with known COVID-19 pneumonia, proved by RT-PCR and referred for CT chest for assessment of the pulmonary changes.

Non-contrast high-resolution chest CT examinations, using (PHILIPS; Ingenuity 8 multislice CT scanner; USA) were done for all patients. All patients were placed in a supine position with both arms elevated. Scan direction was caudocranial in all patients. Scout was taken starting from 1 cm below the lowest costo-phrenic angle to 1 cm above the lung apices.

The CT parameter of the CT chest without contrast as follows: The 1.25 mm thickness, 0.625 mm interval using 512 × 512 matrix with tube speed 35 mm/rotation and 0.5 s rotation time. The KV was 120 and mA ranging from 150 to 400 according to the body weight.

A radiologist with 5 years of experience in CT chest examinations has performed the analysis of CT findings. A CT scoring system was used to quantitatively estimate the pulmonary involvement, depending upon the area of pulmonary affection. To calculate the CT-SS, both lungs were first divided into 5 lobes. Then, each lobe was scored on a scale 0–5 by visual inspection. The score 0 indicated no affection. Score 1 indicated less than 5% affection, score 2 indicated 5–25% affection, score 3 indicated 26–49% affection, score 4 indicated 50–75% affection, and score 5 indicated more than 75% affection. The sum of individual lobar scores was done to calculate the total CT severity score which was ranging from 0 (no affection) to 25 (maximum affection).

All CT images were viewed using a PHILIPS workstation, with multi-planar reformation. Written informed consent was obtained from all patients, and the study protocol was approved by the institutional research ethics committee.

Unilateral diaphragm paralysis is often first suspected during the CT chest examination after the finding of an abnormally elevated hemidiaphragm, which can be defined as a right hemidiaphragm sitting > 2 cm higher than its left counterpart or a left hemidiaphragm sitting equal or higher than the right hemidiaphragm.

The first step to confirm the diagnosis of uni-lateral diaphragmatic paralysis was the exclusion of possible alternative explanations for this finding. Among these, congenital diaphragmatic hernias, atelectasis of various causes, pulmonary and diaphragmatic masses as well as intra-abdominal processes, such as ascites or subphrenic masses were excluded. Another common finding that could suggest uni-lateral diaphragmatic paralysis was atelectasis at the base of the lung on the affected side.

The second step was to evaluate that the diaphragmatic copula was homogenous, regular, and continuous, with no ipsilateral retraction.

The third step was to confirm the diagnosis of uni-lateral diaphragmatic paralysis using Fluoroscopy, using sniff test for 23 cases with suspected uni-lateral diaphragmatic paralysis on CT chest examination.

The sniff test is a quick and easy real-time assessment of diaphragmatic movement. It can be done by asking the patient to practice sniffing before the study with the patient either standing (preferred) or at a supine position, breathing quietly through an open mouth, in front of fluoroscopy. Then, the patient was asked to take few quick short breaths in with a closed mouth (“sniffs”) causing rapid inspiration.

In normal diaphragmatic motion, both hemi-diaphragms move together up and down during expiration and inspiration respectively. In healthy patients, 1–2.5 cm of excursion is normal in quiet breathing.

In-unilateral diaphragmatic paralysis, the affected hemidiaphragm does not move downwards during inspiration and paradoxical movement can occur.

During routine follow-up of the pulmonary changes of COVID-19, complete recovery of the phrenic paralysis was suggested when there was a restoration of the normal position of the affected diaphragmatic copula and the two hemi-diaphragms were almost at the same level.

The Statistical Package for the Social Sciences (version 24; IBM Corp., Armonk, NY, USA) was utilized in the data manipulation and significance testing. The chi-square correlation analysis was conducted, and p values of < 0.1 were used to denote statistical significance.

## Results

Our study included 1527 patients [953 males (62.4%) and 574 females (37.6%)] with ages ranging between 22 to 86 years (mean age of 58 ± 5.2 years with positive RT-PCR for COVID-19 pneumonia (Table 1).

**Table 1:** Demographic features of the study population

Characteristics	Number
**Age**	22 to 86 years (mean age of 58 ± 5.2 years)
**Gender**	
Males	953 males (62.4%)
Females	574 females (37.6%)

The patients with age group between 58 and 86 years (778 patients; 50.9%) were the most affected group. The second affected age group was between 28 and 58 years age group (687 patients; 45%), then the < 28 years age group (62 patients; 4.1%). There was a significant correlation between the disease severity and age group 58–86 years old (p = 0.031).

The CT-SS value was ranging from 1 up to 25, with a mean value of 10.8 and a median value of 12.5. The cut-off value of the CT severity score between the mild and severe cases was 15 with 81.3% sensitivity and 91% specificity. 42.5% of the patients had CT-SS of 11–15. The mild group (CT-SS of 1–15) included 1374 patients, whereas the severe group (CT-SS of 16–25) was composed of 152 patients (Table 2).

**Table 2:** Correlation between CT-SS value and the common CT chest findings in patients with COVID-19 pneumonia

Location	Number and percentage of cases	Significance (***p*** value)
Unilateral	115 (7.5%)	0.223
Bilateral	1412 (92.5%)	0.235
More severity score for lower lobes	910 (59.6%)	0.023
More severity score for upper lobes	198 (12.9%)	0.652
Same severity score for upper and lower lobes	407 (26.6%)	0.058
More severity score for middle lobe/lingula	12 (0.8%)	0.602

Regarding the predominant disease distribution, we found that COVID-19 has typical peripheral and predominant sub-pleural distribution, with lower lobar predilection and bilateral involvement (92.5%). Also, there was a higher CT severity score for the lower lobe affection which was observed in 59.6% of the patients (Table 3).

**Table 3:** The CT-SS value and the corresponding number of patients with COVID-19 pneumonia

CT severity score	Number of cases
1–5	393 (25.7%)
6–10	297 (19.4%)
11–15	684 (44.8%)
16–20	112 (7.4%)
21–25	41 (2.7%)

We had found 23 patients (about 1.5%) presented with incidental unilateral diagrammatic paralysis (14males and 9 females). No cases were found with bilateral diaphragmatic paralysis. 12 patients (52.2%) with unilateral diaphragmatic paralysis had a mild form of the disease process with CT-SS from 1 to 15, while 11 patients (47.8%) had a severe form of the disease process with CT-SS from 16 to 25. No significant correlation between unilateral diaphragmatic paralysis and CT-SS with p value = 0.28.

**Fig. 1:**
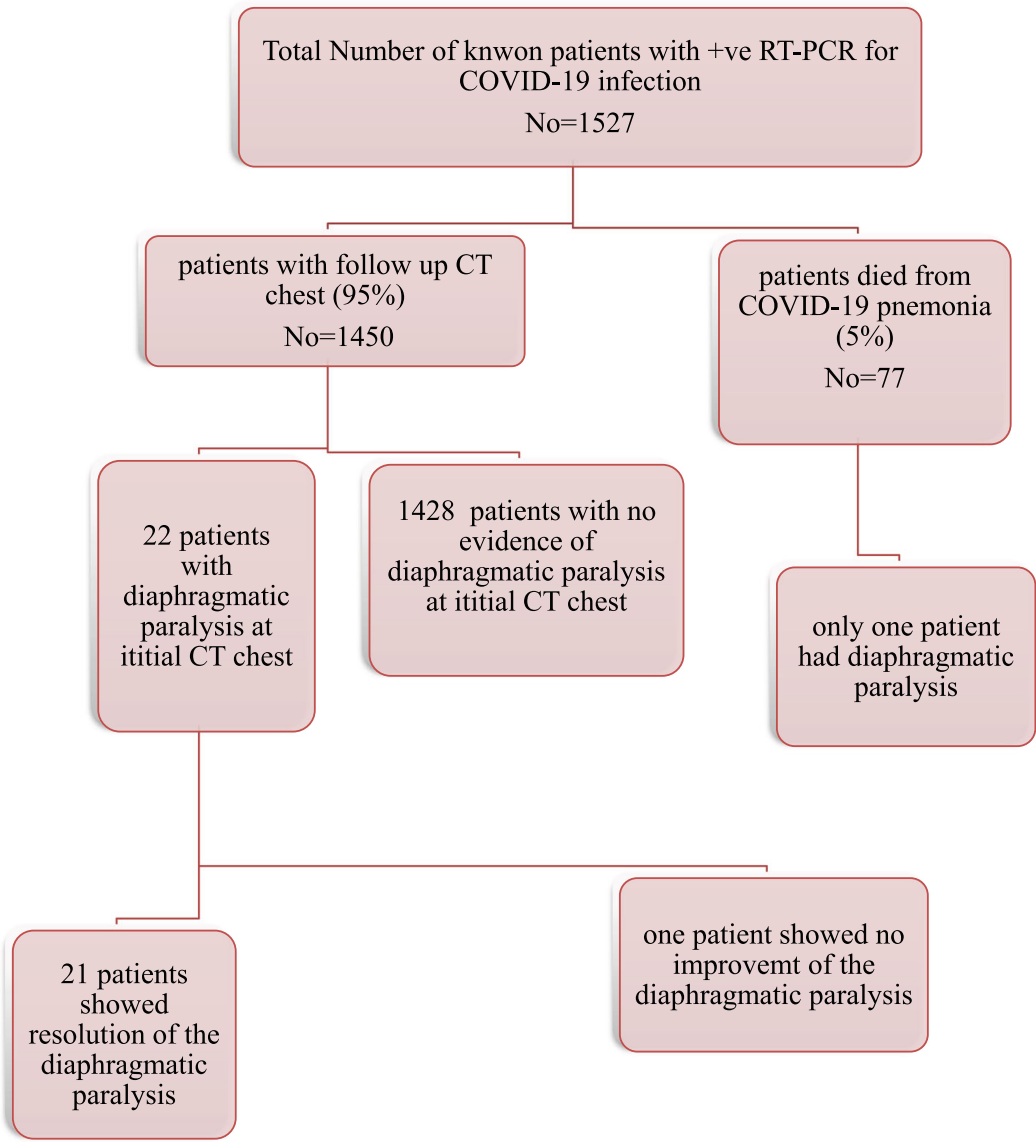
Algorithm representing the demographic data and study methodology

Among the 23 patients with unilateral diaphragmatic paralysis, one patient died from respiratory failure, and representing 0.07% of all cases died with COVID-19 pneumonia. The remaining 22 patients underwent close follow up and the phrenic paralysis was assessed during routine follow-up for pulmonary changes. Twenty-one patients have shown complete recovery of the phrenic paralysis and one patient still has unilateral phrenic paralysis after 2 months from the initial diagnosis (Fig. 1).

## Discussion

The clinical presentation and the pulmonary involvement of COVID-19 are heterogeneous, with a wide spectrum from asymptomatic cases up to respiratory failure [14–17].

Ran et al. found that the CT-SS was higher in severe cases when compared with mild cases. Moreover, the CT-SS threshold of 19.5 could identify severe cases with COVID-19 pneumonia, with a sensitivity of 83.3% and a specificity of 94%, resulting in a negative predictive value of 96.3%. They reported that this easy and available method could provide objective means to identify the patients with severe disease, especially in situations of limited availability of health care resources [18, 19].

In addition, Francone et al. have stated that CT-SS of ≥ 18 is highly predictive of poor prognosis with increased risk of mortality in short-term follow-up. Therefore, they reported that the CT severity score of 18 was considered the cutoff value to differentiate between the mild and severe cases [20]. In our study, we found that the cut–off value of CT severity score between the mild and severe cases was 15 with 81.3% sensitivity and 91% specificity.

We have found that COVID-19 has typical peripheral and sub-pleural distributions, with lower lobar predilection and bilateral involvement. Also, there were higher CT severity scores for the lower lobes with an insignificant p value. These findings were similar to the study by Salehi et al. who have reported that CT features of COVID-19 infection had predominant bilateral involvement and peripheral distribution in 87.5% and 76.0% of their patients, respectively [21]. Also, Zhou et al. have reported that 77.4% of the patients had a predominantly peripheral distribution of lesions; and the mean CT-SS for middle and lower zones was significantly higher than that for the upper lung zone, and no significant difference in the mean CT-SS was observed between the middle and lower zones [22].

We have incidentally discovered 23 patients with unilateral phrenic paralysis during the initial evaluation of patients with positive RT-PCR for COVID-19 pneumonia. Twelve patients with phrenic paralysis had a mild form of the disease process with CT-SS from 1 to 15, while 11 patients had a severe form of the disease process with CT-SS from 16 to 25. We had 21 patients showing almost complete recovery of the phrenic paralysis, and one patient still had residual phrenic paralysis despite improvement of the pulmonary changes on CT examination. One patient with unilateral phrenic paralysis died from respiratory failure. We suggest this was caused by direct peripheral neurological involvement of phrenic nerves. Virologists have described neurological lesions causing anosmia and dysgeusia, which were not recognized in patients in Wuhan [23]. Moreover, mono-neuritis multiplex, meningoencephalitis, and myelitis were thought to result from a direct viral attack on the nerve cells and were described as a result of SARS-CoV-19 neurotropism [24]. Increasing evidence also shows that SARS-CoV-19 may invade peripheral nerve terminals. This observation suggests the virus may cause phrenic paralysis in COVID-19 patients, resulting in pulmonary decompensation [25].

Phrenic nerve paralysis could be suggested in patients with orthopnea and paradoxical abdominal respiration. Phrenic paralysis is considered a rare neurological complication of COVID-19 which may aggravate pulmonary decompensation.

**Fig. 2:**
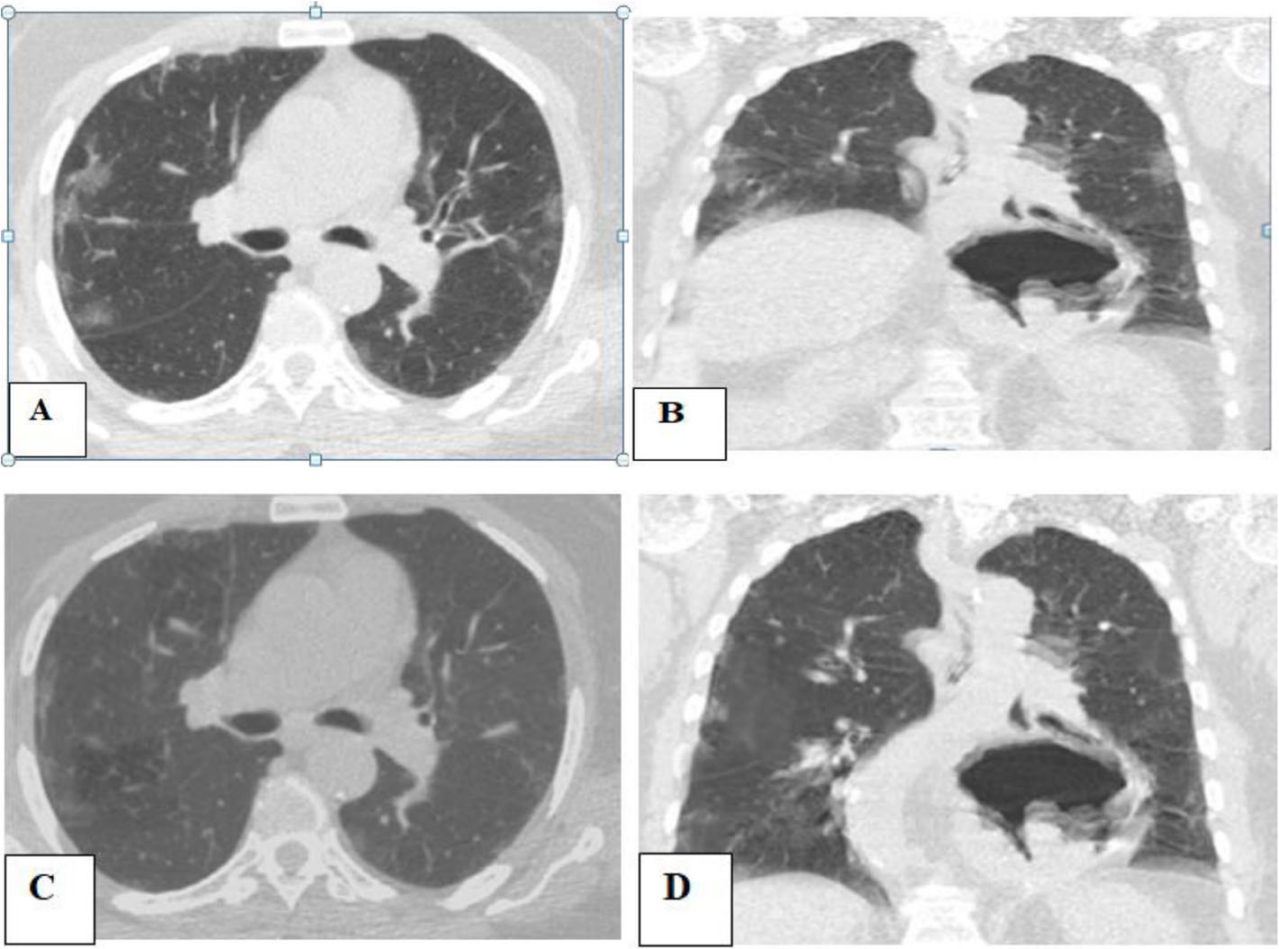
Fifty-one-year-old male patient, with a history of COVID-19 pneumonia, referred for initial CT evaluation with CT severity score 8. **A**, **B** Axial and coronal CT chest imaging respectively shows bilateral sub-pleural ground-glass patches and pulmonary consolidation with interlobar septal thickening. Incidental discovery of right-sided diaphragmatic paralysis. A notice of large axial hiatus hernia. **C**, **D** Images after 4 weeks reveals remarkable regression of bilateral patchy ground glassing and consolidation with resolution of the previous right phrenic paralysis

**Fig. 3:**
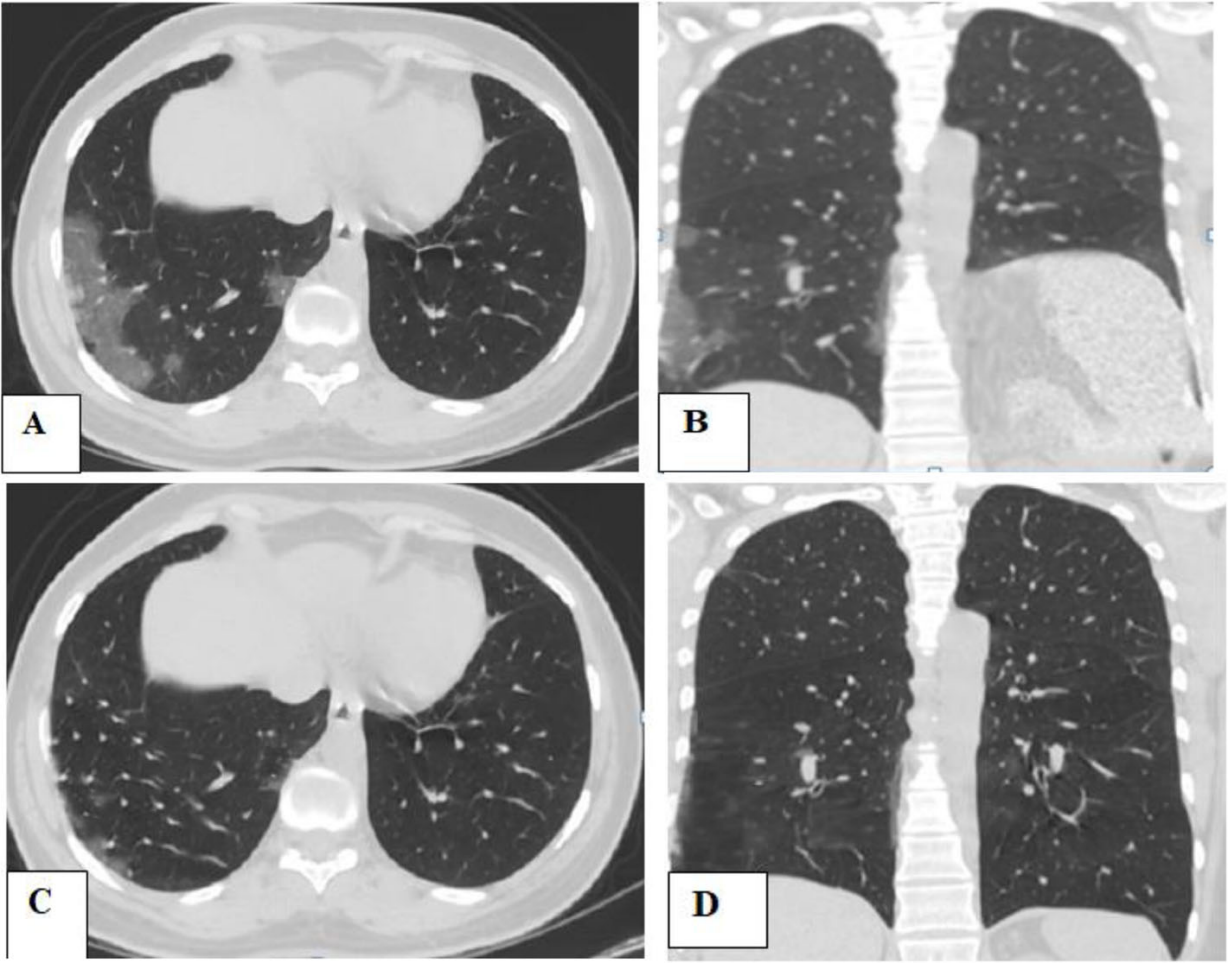
Seventy-eight-year-old male patient, with a history of COVID-19 pneumonia, referred for initial CT evaluation with CT severity score 7. **A**, **B** Axial and coronal CT chest imaging respectively showed right sub-pleural ground-glass patches and pulmonary consolidation with interlobar septal thickening. Incidental discovery of left-sided diaphragmatic paralysis. **C**, **D** Images after 4 weeks reveals remarkable regression of the right-sided patchy ground glassing and consolidation with resolution of the previous left phrenic paralysis

**Fig. 4:**
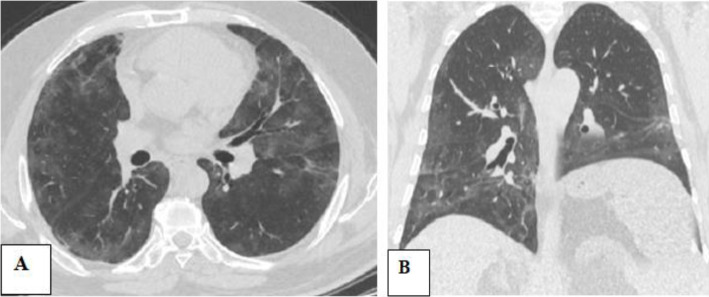
Seventy-eight-year-old male patient, with a history of COVID-19 pneumonia, referred for initial CT evaluation with CT severity score 21. **A, B** Axial and coronal CT chest imaging respectively shows bilateral sub-pleural ground-glass patches and pulmonary consolidation with interlobar septal thickening. Incidental discovery of left-sided diaphragmatic paralysis. The patient died after 2 weeks from respiratory failure

The main limitation of this study was the small number of cases incidentally discovered with unilateral phrenic paralysis, although the large sample size of our study. Contribution from multi-center and sharing experience with other countries will be of additive value to better assess such rare but serious complications of COVID-19 pneumonia (Figs. 2, 3, and 4).

## Conclusion

Phrenic paralysis is considered a serious but rare neurological complication of COVID-19 pneumonia. No correlation between the CT severity score and the incidental discovery of unilateral diaphragmatic paralysis. The possibility of neurological disorder caused by SARS-CoV-2 on peripheral nerves, especially the vagus nerve, should be determined by the investigation. Its association with anosmia, dysgeusia, other neurological symptoms, should be considered.

## Data Availability

All the datasets used and analyzed in this study are available with the corresponding author on reasonable request.

## Abbreviations

COVID: Coronavirus disease
MERS-CoV: The Middle East respiratory syndrome coronavirus
CT: Computed tomography
CT-SS: Computed tomography severity score
SARS: Severe acute respiratory syndrome
RT-PCR: Reverse transcription-polymerase chain reaction
